# A Systematic Review of the Potential of *Acmella* Genus Plants for the Treatment of Musculoskeletal Disorders

**DOI:** 10.3390/ijms26136493

**Published:** 2025-07-05

**Authors:** Mohd Maaruf Abdul Malik, Ahmad Nazrun Shuid, Nurul Raudzah Adib Ridzuan, Isa Naina Mohamed, Elvy Suhana Mohd Ramli, Ahmad Naqib Shuid, Rohanizah Abdul Rahim

**Affiliations:** 1 Faculty of Dentistry, Universiti Teknologi MARA, Sungai Buloh Campus, Jalan Hospital, Sungai Buloh 47000, Malaysia; maaruf@uitm.edu.my; 2 Faculty of Medicine, Universiti Teknologi MARA, Sungai Buloh Campus, Jalan Hospital, Sungai Buloh 47000, Malaysia; nraudzah@uitm.edu.my; 3 Faculty of Medicine, Universiti Kebangsaan Malaysia Medical Centre, Jalan Yaacob Latif, Cheras 56000, Malaysia; elvysuhana@ukm.edu.my; 4 Advanced Medical and Dental Institute, Universiti Sains Malaysia, Bertam, Kepala Batas 13200, Malaysia; naqib@usm.my; 5 Institute for Research in Molecular Medicine, Universiti Sains Malaysia, Pulau Pinang 11800, Malaysia; rohanizah@usm.my

**Keywords:** *Acmella* genus plant, musculoskeletal disorders, osteoporosis, muscle strains, tendonitis, osteoarthritis

## Abstract

The genus *Acmella* has received growing attention for its pharmacological properties, including its potential applications in musculoskeletal disorders (MSDs). Plants in this genus, such as *Spilanthes acmella*, *Blainvillea acmella*, *Acmella uliginosa*, and *Acmella oleracea* contain various bioactive compounds which have demonstrated anti-inflammatory, analgesic, and anti-arthritic properties. This systematic review evaluates the clinical and preclinical evidence supporting the use of plants from *Acmella* genus for the treatment of MSD, such as arthritis, osteoporosis, muscle injuries, joint inflammation, and other related pathologies. The methodology used in this study involved a systematic literature review, following the Preferred Reporting Items for Systematic Reviews and Meta-Analysis (PRISMA) guidelines, along with synthesis analysis and quality appraisal. The articles were retrieved from Scopus, Google Scholar, and PubMed databases. Eleven articles were further analyzed to determine the therapeutic potential of *Acmella* genus plants for musculoskeletal disorders. The plants included were *Spilanthes acmella, Blainvillea acmella, Acmella uliginosa,* and *Acmella olerecia.* The musculoskeletal disorders investigated were osteoporosis, osteoarthritis, and myopathies. The extracts from these plants were shown to decrease inflammation, enhance joint health, relieve pain, and stimulate osteogenic activity. These effects may be attributed to several active compounds found in these plants. The available evidence suggests that *Spilanthes acmella* and *Blainvillea acmella* have the potential to treat osteoporosis. *Acmella oleracea* and *Acmella uliginosa* have the potential to be used for the treatment of osteoarthritis, while *Spilanthes acmella* is used to treat myopathies. Further research is needed to establish the efficacy, optimal dosing, and safety of these plants.

## 1. Introduction

Medicinal plants have long been a primary and widely used source of safe and effective remedies among populations. Although the use of synthetic medicines has grown in recent years [1,2], the World Health Organization (WHO) estimates that approximately 80% of the global population continues to rely on medicinal plants as a primary means of addressing health issues, particularly in developing countries [3]. Musculoskeletal disorders are a significant global public health concern, impacting the muscles, bones, joints, tendons, ligaments, and related structures. Conditions such as arthritis, osteoporosis, tendonitis, and myopathies are prominent contributors to disability and diminished quality of life.

The Global Burden of Disease study estimates that over 527 million individuals are affected by osteoarthritis worldwide [4,5]. The prevalence of osteoarthritis was predicted to rise as the population ages [6]. More than one-third of individuals over the age of 65 have osteoarthritis in at least one joint, reinforcing its classification as an age-related condition predominantly affecting the elderly population [7]. In addition, it was discovered that the prevalence of inflammatory myopathies ranged from 2.4 to 33.8 per 100,000 people, while the incidence rate varied from 1.16 to 19/million/year [8].

Numerous variables can contribute to the development of osteoarthritis, such as genetics, sports, obesity, past joint injuries, occupational strain, or anomalies in joint architecture [9,10,11]. These factors may also influence the onset of osteoarthritis in younger individuals [12,13,14]. While pharmacological treatments like nonsteroidal anti-inflammatory drugs, corticosteroids, and disease-modifying antirheumatic drugs are commonly used, their effectiveness is often constrained by side effects, highlighting the need for alternative therapeutic approaches [6,15,16,17].

Plant-derived natural products are increasingly recognized for their potential in addressing pain, inflammation, and other musculoskeletal conditions. Within traditional medicine, species from the Asteraceae family have been extensively utilized for their pain-relieving and anti-inflammatory effects [18]. Notably, plants from the *Acmella* genus, including *Spilanthes acmella*, *Blainvillea acmella, Acmella oleracea*, and *Acmella uliginosa*, are being actively studied for their bioactive properties.

The objective of this systematic review is to summarize the evidence on the use of *Acmella* genus plants, specifically *Spilanthes acmella*, *Blainvillea acmella*, *Acmella uliginosa*, and *Acmella oleracea*, for musculoskeletal disorders by evaluating preclinical and clinical evidence.

### Background on Acmella Genus Plants

*Spilanthes acmella*, *Blainvillea acmella*, *Acmella uliginosa*, and *Acmella oleracea* are all plants that belong to the Asteraceae family, commonly known as the daisy family or sunflower family. These plants are typically herbaceous and perennial, often growing in tropical and subtropical regions. They generally have yellow or orange flower heads with a daisy-like appearance. They are also called toothache plant, buzz button, or paracress, depending on cultural or regional naming conventions [19,20].

This family includes a wide variety of plants, many of which are used in traditional medicine for their anti-inflammatory, antibacterial, and analgesic properties. The primary compounds of interest include spilanthol and other alkylamides and flavonoids, contributing to their medicinal properties [21,22,23,24]. According to a bioassay-guided fractionation, spilanthol was shown to be the active ingredient in *Acmella oleracea*’s anti-inflammatory effects on macrophages, partly due to a reduction in NF-κB activation. Additionally, there was no noticeable cytotoxic effect after incubating spilanthol with LPS-stimulated human neutrophils, although there was a notable decrease in the release of cytokines (IL-8 and TNF-α) [25].

They are all related through the *Acmella* genus, but the species within this genus are sometimes grouped under different scientific names. The confusion in the classification and naming of herbal plants results from several factors, including taxonomic changes, variations in common names across regions and cultures, morphological variability, and a lack of standardization in classification systems [26,27].

To avoid confusion, this systematic review addressed all these plants as *Acmella* genus plants based on authoritative sources such as Kew’s Plants of the World Online (POWO) and International Plant Names Index (IPNI) [28] (Table 1). In search of relevant journal articles, the keyword ‘Acmella’ was used to retrieve all the articles on related plants such as *Spilanthes acmella*, *Blainvillea acmella*, *Acmella uliginosa*, *Acmella oleracea*, and *Acmella caulirhiza*. The keyword ‘spilanthol’ was also used since it is the active compound for the *Acmella* genus plants.

## 2. Materials and Methods

### 2.1. Search Strategy

This systematic review analyzed all published studies investigating the musculoskeletal diseases of plants from the *Acmella* genus, including *Spilanthes acmella*, *Acmella oleracea*, *Acmella paniculata*, *Acmella uliginosa*, and *Acmella caulirhiza*. The selection of eligible studies was guided by the PICOS framework (Population, Intervention, Comparison/Comparator, Outcomes, and Study design) [29] (Table 2). The goal was to identify relevant studies investigating the therapeutic effects of plants from *Acmella* genus in treating musculoskeletal disorders. A systematic review of the literature was conducted across Scopus, Google Scholar, and PubMed databases to identify the relevant studies.

The search strategy to identify relevant articles involved using the following keywords: *Acmella*; spilanthol; musculoskeletal; arthritis; osteoarthritis; tendonitis; osteoporosis; bone; muscle. These keywords were combined using Boolean operators such as AND, OR, and parentheses to ensure logical grouping and exhaustive retrieval (Table 3).

In the screening phase, the articles were selected according to the inclusion and exclusion criteria (Table 4).

### 2.2. Eligibility of Research Articles

Articles to be included in the review were selected in three phases. First, the titles of the articles were screened, and any article that did not match the inclusion criteria was excluded. During this phase, duplicates were also removed. Second, the abstracts of the remaining articles were screened and excluded if they did not meet the inclusion criteria. Lastly, the full text of the remaining articles from the second phase was obtained. The selected articles were thoroughly read to exclude any articles that did not meet the inclusion criteria.

The screening was carried out by at least two reviewers. The selection of articles to be included in the review had to be agreed upon by at least two reviewers before proceeding to the data extraction phase. Any discrepancies were resolved through consensus between the reviewers.

### 2.3. Data Extraction

Data extraction was performed in a standardized manner with the use of a data collection form. Extracted data included the following: (1) authors; (2) type of plants and dose; (3) study design and sample size; (4) objectives; (5) parameters; (6) findings, and (7) conclusion. Extracted data were tabulated to facilitate comparative analysis.

### 2.4. Quality Assessment

To ensure the reliability of the included studies, their quality was assessed by two independent researchers using standardized evaluation tools. The Newcastle-Ottawa Scale (NOS) was applied to evaluate the quality of human interventional studies, while SYRCLE’s risk of bias tool was used for in vivo and in vitro studies, assessing biases such as selection, performance, detection, attrition, and reporting. Studies deemed to be of low quality were either excluded from the review or their limitations were explicitly acknowledged.

### 2.5. Data Synthesis

The data synthesis was carried out using a narrative synthesis approach. Preclinical studies (including in vitro and in vivo models) were analyzed separately from clinical trials due to variations in methodologies and outcome measures. The process involved the following steps:

- Summarizing preclinical findings related to mechanisms affecting inflammation, bone formation, and joint health.
- Assessing clinical evidence with a focus on key musculoskeletal outcomes, such as pain reduction, decreased joint inflammation, and functional improvement.
- Investigating shared mechanisms of action observed in both clinical and preclinical studies, particularly the effects of compounds like spilanthol on pathways such as nuclear factor kappa-light-chain-enhancer of activated B cells (NF-κ), Wnt/β-catenin, and others involved in bone metabolism.

### 2.6. Handling Missing Data

For studies with incomplete or missing data, efforts were made to reach out to the corresponding authors to request clarification or supplementary information. If no response was obtained, the study was either excluded or analyzed solely using the data that were available.

### 2.7. Ethical Considerations

While this systematic review did not involve direct experimentation on humans or animals, it adhered to ethical research standards. The included studies were all peer-reviewed publications that had obtained ethical approval for their respective experiments and clinical trials.

## 3. Results

The literature searches on the databases identified 322 relevant articles. Two reviewers independently assessed the titles and abstracts of all articles for inclusion or exclusion criteria. Differences in opinion between the reviewers regarding the selection of the articles were resolved by discussion. A total of 18 articles were retrieved for further assessment of their full texts. Seven of these articles were excluded because the interest of this review was not part of their primary studies, or they were not related to the objective of the systematic review.

**Figure 1:**
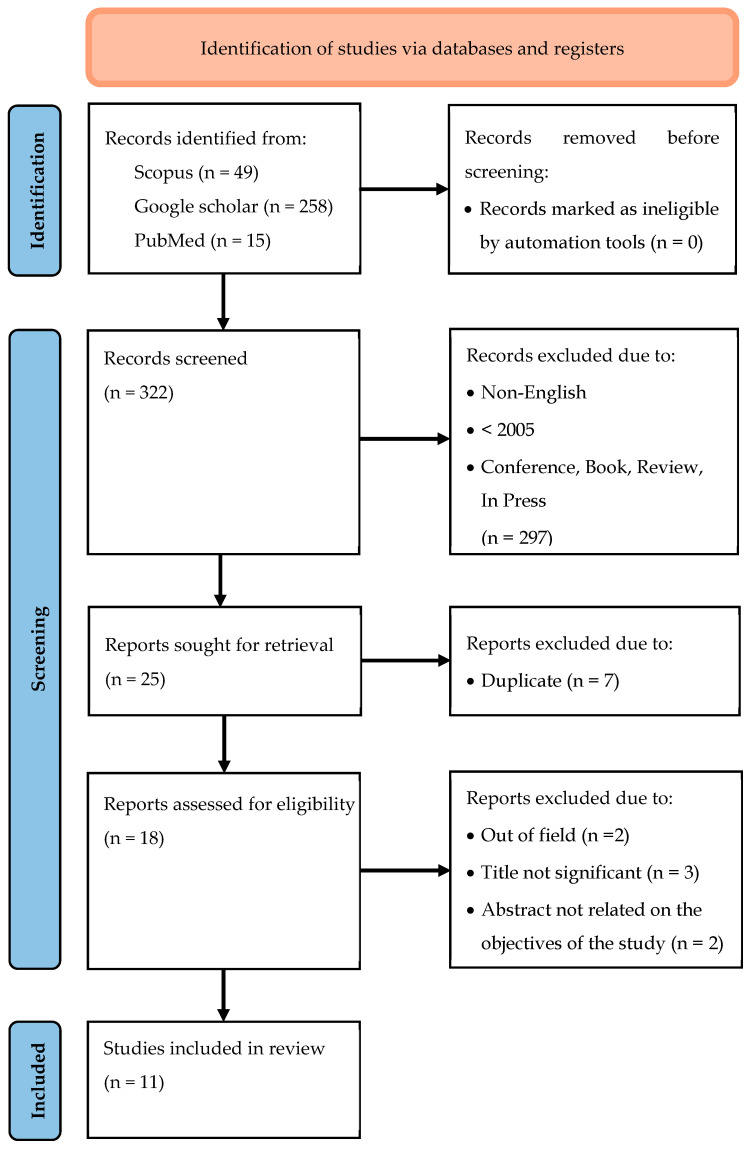
Prisma 2020 flow diagram.

In terms of quality assessments, all the selected articles have a low risk of bias. For the human studies by Rondanelli et al. [30] and Pradhan et al. [31], by using the Newcastle-Ottawa Scale (NOS), the quality of these interventional studies was found to be acceptable. While for the rest of the studies, SYRCLE’s risk of bias tool did not detect any major biases. Finally, 11 articles were included for the purpose of this review. A flow chart of the selection process, including reasons for exclusion, is shown in Figure 1.

### 3.1. Overview of Evidence

#### 3.1.1. Effects on Bone

There are four studies selected in relation to the effects of *Acmella* genus plants on bone cells, with three in vitro studies and one in vivo study. As for the in vitro studies of Widyowati et al. [32] and Abdul Rahim et al. [33], MC3T3-E1 cells (osteoblast-like cells) were treated with either ethanol or methanol extract of *Spilanthes acmella* or *Blainvillea acmella* leaves at doses ranging from 2.93 µg/mL to 1500 µg/mL. In another study by Widyowati et al. [34], MC3T3-E1 cells were exposed to 12 isolated compounds of *Spilanthes acmella* at doses of 12.5 and 25 μM.

All the in vitro studies had measured alkaline phosphatase (ALP) activities to determine bone formation activities by osteoblasts. In addition, Widyowati et al. [34] had measured calcium deposition, while Abdul Rahim et al. [33] measured calcium deposition and collagen formation as well. All the studies have concluded that *Acmella* genus plants were able to promote bone formation. Widyowati et al. [34] have also identified six compounds responsible for bone formation. Abdul Rahim et al. [33] managed to positively correlate the phenolic contents of the plants with the bone formation activities.

With regard to the in vivo study using a steroid-induced osteoporosis mouse model, the combination of the plant with exercise increased the osteoblast cells [35]. Overall, it can be concluded that the leaf extract of this plant exhibited bone formation activities in both in vitro and in vivo studies. Therefore, it may be investigated further as an anti-osteoporotic agent, especially in terms of animal and human studies.

#### 3.1.2. Effects on Muscle

There is only one study by Pradhan et al. [31] investigating the effects of *Acmella* genus plant on muscle mass. It was a human study carried out on 240 subjects receiving commercial preparations of *Spilanthes acmella* (SA3X) for 2 months. Muscle mass was assessed by measuring the circumferences of mid-upper arm (MUAC), chest (CC), and thigh (TC). It was found that *Spilanthes acmella* had caused a significant increase in mid-upper arm circumference. There were no significant changes in the circumferences of the chest and thigh. Thus, this indicates that *Spilanthes acmella* may be used to stimulate muscle growth.

#### 3.1.3. Effects on Joint

The studies were carried out to determine the anti-inflammatory activities of *Acmella* genus plant extracts in treating osteoarthritis. The inclusion criteria of this review were satisfied by one in vitro study, five in vivo studies, and one human study. Stein et al. [36] had carried out both in vitro and in vivo studies. In the in vitro study, *Acmella oleracea* extract and spilanthol treatments on Vascular Smooth Muscle Cells (VSMCs) in hyperglycemic media were able to reduce chymase activity and expression and reduce reactive oxygen species.

As for the in vivo studies, the leaves or flowers extracts of *Spilanthes acmella*, *Acmella uliginosa*, and *Acmella oleracea* were used, mostly given orally to the animal model, except for Stein et al. [36], who injected the extract intraperitoneally, and Moro et al. [37], who applied the extract topically to the site of tendon injury. Furthermore, Stein et al. [36] had isolated spilanthol and used it as one of the treatments.

Doses of *Acmella* genus plant extracts given orally or intraperitoneally to animal models varied from 10 to 833 mg/kg. For the topical application by Moro et al. [37], *Acmella oleracea* lyophilizate was added to a base ointment of anhydrous lanolin and solid Vaseline 30:70 *w*/*w* at a concentration of 20% *w*/*w*. Several types of irritants were used to induce paw edema or arthritis in rats, such as Carrageenan and Freund’s Complete Adjuvant (CFA) [38] as well as monosodium iodate [39]. At the same time, Moro et al. [37] induced arthritis surgically by partial transection of the calcaneal tendon.

In all the in vivo studies, the *Acmella* genus plant extracts were able to reduce pain, swelling, and inflammation, with improved joint histology. Biochemically, the extracts were also able to reduce inflammatory cytokines (interleukin-1 beta; IL-1β and tumor necrosis factor; TNF-α), nitric oxide (NO), and creatinine levels, while hemoglobin, serum protein, and albumin levels were raised. In the animal study by Paul et al. [40], a combination of *Acmella uliginosa* and aloe vera was found to produce synergistic anti-arthritis and anti-inflammatory actions. Paul et al. [40] were also able to identify five potent anti-inflammatory compounds within *Acmella uliginosa,* namely 9-Octadecenoic acid (Z)-phenylmethyl ester, à-N-Normethadol, astaxanthin, caryophyllene oxide, and fenretinide.

Nevertheless, in the human study by Rondanelli et al. [30], food-grade lecithin-based formulation of *Zingiber officinale* (ginger) and *Acmella oleracea* standardized extracts (Mitidol™) in the form of a tablet was given orally to 50 patients with knee osteoarthritis, twice daily for 4 weeks. The combination of *Acmella oleracea* and *Zingiber officinale* was effective in reducing joint pain as shown by the reduced visual analog scale (VAS), improved knee function as indicated by better Western Ontario and McMaster Universities Osteoarthritis (WOMAC) index and Tegner Lysholm Knee Scoring Scale, and improved 36-Item Short Form Health Survey (SF-36) physical activity and dual-energy X-ray absorptiometry (DEXA) fat distribution. This was accompanied by a reduction in inflammatory markers, erythrocyte sedimentation rate (ESR), and C-reactive protein (CRP).

Table 5 shows a brief characteristics and summary of the information retrieved from the 11 studies that fulfilled the inclusion criteria and were incorporated into the review.

## 4. Discussion

Musculoskeletal disorders (MSDs) encompass a range of conditions that impact the muscles, bones, joints, tendons, ligaments, and other structures that provide support and enable movement. These disorders can result in pain, stiffness, inflammation, and reduced mobility, greatly affecting a person’s capacity to carry out everyday tasks. Common examples of MSDs include arthritis, osteoporosis, back pain, tendonitis, and muscle strains. These conditions may arise from various factors, such as injuries, repetitive strain, aging, or underlying health issues [41,42].

The *Acmella* genus consists of flowering plants belonging to the Asteraceae family, known for their medicinal and bioactive properties. Species like *Spilanthes acmella* and *Acmella oleracea* contain bioactive compounds, such as spilanthol, which are thought to contribute to their therapeutic effects [43,44]. In this review, the potential therapeutic effects of *Acmella* genus plants on MSD were assessed.

Agents that promote bone formation or bone anabolic activity have the potential to be developed as drugs for the prevention or treatment of osteoporosis [45,46,47]. To date, studies looking at the potential of *Acmella* genus plants in the prevention and treatment of osteoporosis are mainly in vitro studies. There is only one in vivo study and no human studies.

The in vitro studies were carried out using MC3T3-E1 as osteoblast-like cells. In these studies, *Acmella* genus plant extracts exhibited bone formation activities and, therefore, have potential as anti-osteoporotic agents. Osteoporosis occurs when there is an imbalance between bone formation and resorption. Besides the bone formation activity by osteoblasts, bone resorption by osteoclasts plays an important role in the pathogenesis of osteoporosis [48,49,50]. In the future, osteoclasts should also be studied to determine whether *Acmella* genus plants may also inhibit osteoclast activity.

The only animal study on *Acmella* genus plants adopted a steroid-induced osteoporosis rat model. Although this is an important type of osteoporosis, the study on postmenopausal osteoporosis, the major type of osteoporosis represented by the ovariectomised rat model, is lacking [51,52,53]. The *Spilanthes acmella* leaf extract given orally at a dose of about 200 mg/kg was successful in maintaining osteoblast number but only when combined with another intervention, exercise. The dose used was low compared to the 1500 mg/kg of *Acmella uliginosa*, another *Acmella* genus plant, which did not produce any toxic effects on rats [37]. In a toxicity study using the zebrafish embryo test, *Spilanthes acmella* showed no lethal effects at the highest tested concentration of 20% *v*/*v*, while 10% *v*/*v* was the lowest concentration that produced a noticeable sublethal effect. Furthermore, the crude extract of *Spilanthes acmella* Linn. Murr., when incorporated into animal feed at concentrations of 0.01% *v*/*v* and 1% *v*/*v*, did not result in any lethal, sublethal, or malformation effects [54].

There should be more animal studies looking at other osteoporosis models, especially estrogen-deficient or postmenopausal models. The sole intervention of the *Acmella* genus plants should be investigated, and more robust bone parameters such as bone histomorphometry, DEXA, and micro-computed tomography (micro-CT) should be employed. If there are positive outcomes with animal studies, then a human study may be warranted.

A study conducted by Widyowati et al. [34] has identified six active compounds in *Spilanthes acmella* which promote bone formation, namely 1,3-butanediol 3-pyroglutamate, 2-deoxy-d-ribono-1,4-lactone, methyl pyroglutamate, ampelopsisionoside, icariside B1, and benzylα-l-arabinopyranosyl-(1→6)-β-d-glucopyranoside. Abdul Rahim et al. [33] have reported that terpenoids of α-cubebene, caryophyllene, caryophyllene oxide, phytol, and flavonoids of pinostrobin and apigenin were the compounds that may contribute to both antioxidant and bone anabolic activities of *Blainvillea acmella*. However, these active compounds should also be tested in animal studies, rather than using the crude extracts. This is important for the development of the compounds isolated from *Acmella* genus plants into anti-osteoporotic agents.

In comparison to the studies on bone, studies on the effects of *Acmella* genus plants on osteoarthritis are more comprehensive, with many animal studies and one human study. *Acmella* genus plants, mainly in the form of *Acmella oleracea*, were found to reduce inflammation in Vascular Smooth Muscle Cells (VSMCs) and various osteoarthritic animal models. The highest dose of the plant extract used was 833 mg/kg, which was still lower than the LD50 dose of *Acmella uliginosa* of more than 1500 mg/kg [36]. In terms of route of administration, Stein et al. [36] had administered the extract intraperitoneally (IP), which was able to reduce pain and swelling of the paws induced by formalin. As opposed to oral administration, IP administration of plant extract has higher bioavailability and faster action [55]. Another study by Moro et al. [37] applied the extract topically to the surgical site after partial transection of the calcaneal tendon. This method of administration acts locally and does not enter systematic circulation, therefore reducing any adverse events [56].

Moreover, *Acmella* genus plants may also be combined with other plants such as aloe vera and ginger to treat osteoarthritis. The combinations are synergistic as the combined effects produce greater anti-arthritis and anti-inflammatory actions. Several active compounds in *Acmella uliginosa* have been identified with anti-inflammatory activities. They warrant further processes to evaluate, optimize, and test them for potential therapeutic use as anti-osteoarthritis agents.

There were positive outcomes from a human study in patients with knee osteoarthritis [30]. However, it was a quasi-experimental design that lacked random assignment, making it susceptible to biases. *Acmella oleracea* was also combined with another plant, ginger, and therefore, the sole anti-osteoarthritis effects of the *Acmella* genus plant cannot be elucidated. All these make it challenging to confidently attribute the observed outcomes to the effects of *Acmella* genus plants.

Furthermore, a human study has reported that supplementation with a commercial preparation of *Spilanthes acmella* was able to increase the muscle mass of the mid-upper arm [31]. However, no increase in muscle mass was recorded for muscles in the chest and thigh regions. There are questions raised about the selectivity of the plant extract on muscle in certain parts of the body. There are also several limitations identified by the authors, such as the recruitment of the participants was not random and selective to gym-goers, and the 24-hour dietary recall method may not be reliable.

## 5. Conclusions

The *Acmella* genus, particularly species like *Spilanthes acmella* and *Acmella oleracea*, shows promise for managing musculoskeletal disorders, with evidence suggesting its potential in both osteoarthritis and osteoporosis treatment. While in vitro studies demonstrate positive effects on bone formation, further in vivo *studies* are needed, especially in estrogen-deficient or postmenopausal models, to confirm these benefits. The active compounds identified in *Acmella* plants may contribute to their anti-inflammatory and bone-promoting properties, warranting further investigation into their therapeutic potential. Although existing in vitro, in vivo, and clinical studies on osteoarthritis show encouraging results, limitations such as small sample sizes and lack of robust data limit these effects. Therefore, continued in vitro and in vivo investigations into isolated compounds, optimal dosages, and intervention methods are essential to fully realize the therapeutic potential of *Acmella* genus plants in treating musculoskeletal disorders.

## Figures and Tables

**Table 1:** Summary of the accepted names and key synonyms for *Acmella oleracea*.

Accepted Name	Synonyms
*Acmella oleracea* (L.) R. K. Jansen	*Spilanthes oleracea* L., *Spilanthes acmella* (L.) Murray (sensu L.C. Clarke) (powo.science.kew.org, accessed on 24 June 2025) *Pyrethrum spilanthus* Medik. (en.wikipedia.org, accessed on 24 June 2025) *Cotula pyrethraria* L. (en.wikipedia.org) *Bidens fixa* Hook.f. (en.wikipedia.org) *Bidens fervida* Lam. (en.wikipedia.org)
*Blainvillea acmella* (L.) Philipson	*Spilanthes acmella* (L.) L. (powo.science.kew.org) *Acmella linnaei* Cass. (powo.science.kew.org) *Acmella mauritiana* Pers. (powo.science.kew.org) *Bidens acmella* (L.) Lam. (powo.science.kew.org) *Ceratocephalus acmella* (L.) Kuntze (powo.science.kew.org) *Coreopsis acmella* (L.) K.Krause (powo.science.kew.org) *Pyrethrum acmella* (L.) Medik. (powo.science.kew.org) *Verbesina acmella* L. (powo.science.kew.org)

**Table 2:** PICOS framework.

	Inclusion	Exclusion
Population	Cell line Animal model Patients	-
Intervention	*Acmella* genus plant extracts	-
Comparison	Cells not receiving *Acmella* plant extracts Positive and negative control groups	-
Outcome	Bone cell parameters Arthritis scoring Muscle circumferences	-
Study Type	In vitro and in vivo studies, randomized controlled studies, case–control studies, cohort studies	Case reports, editorials, communications, reviews, meta-analysis

**Table 3:** Search syntax used in study.

Source	Search Term	Filters	Number of Results
Scopus	TITLE-ABS-KEY [(“*Acmella*” OR “spilanthol”) AND (musc* OR arth* OR tendon* OR osteo* OR bone)]	English language Publication years: 2004–2024	49
Google Scholar	(“*Acmella*” OR “spilanthol”) AND (musc* OR arth* OR tendon* OR osteo* OR bone)	English language Publication years: 2004–2024	258
PubMed	[(“*Acmella*” [All Fields] OR “spilanthol” [All Fields]) AND (“musc*” [All Fields] OR “arth*” [All Fields] OR “tendon*” [All Fields] OR “osteo*” [All Fields] OR (“bone and bones” [MeSH Terms] OR (“bone” [All Fields] AND “bones” [All Fields]) OR “bone and bones” [All Fields] OR “bone” [All Fields])	English language Publication years: 2004–2024	15

**Table 4:** Selection criteria for papers included in the systematic review.

Inclusion Criteria	Exclusion Criteria
English language	Non-English language articles
Articles published within the past 20 years (2004–2024)	Articles published earlier than 2004
Articles with abstracts	Reviews or meta-analyses
Research articles	Letters, editorials, or case studies

**Table 5:** Characteristics and summary of the studies included in the review.

Author and Year	Study Design	Type of Plant Extract and Its Dosage	Sample Size	Musculoskeletal Related Objective	Parameters	Musculoskeletal Findings	Outcomes
Widyowati et al., 2011 [32]	In vitro study	Ethanol extract of the leaves of *Spilanthes acmella* Dose: 50 μg/mL	MC3T3-E1 osteoblast cells	To discover the ideal anabolic agent by measuring on alkaline phosphatase (ALP) activity as a marker of osteoblast differentiation	i. ALP activity	The *Spilanthes acmella* had a dose-dependent stimulatory activity on ALP up to 25 g/mL	*Spilanthes acmella* has bone anabolic activities
Abdul Rahim et al., 2022 [33]	In vitro study	Ethanol extract of *Blainvillea acmella* leaves Dose: 2.93 µg/mL to 1500 µg/mL	MC3T3-E1 osteoblast cells	To determine the relationship between phytochemical compounds, antioxidants and bone anabolic activities of *Blainvillea acmella*	i. GCMS and LCTOFMS analyses. ii. Antioxidant activities: DPPH, ABTS, and FRAP assays iii. Bone formation: collagen formation, ALP activity, and Alizarin red assay	Positive correlations were observed between phenolic content to antioxidant and bone anabolic activities	*Blainvillea acmella* may be a valuable antioxidant and anti-osteoporosis agent
Widyowati et al., 2020 [34]	In vitro study	Isolated compounds of methanol extract of *Spilanthes acmella* leaves Dose: 12.5 and 25 μM	MC3T3-E1 osteoblast cells	To test the isolated compounds of *Spilanthes acmella* for bone formation activities	i. ALP activities ii. Calcium deposition (Alizarin red staining)	These compounds stimulated both ALP and mineralization activities.	Six active compounds in *Spilanthes acmella* were identified to promote bone formation and mineralisation
Laswati et al., 2015 [35]	In vivo study	Ethanol extract of the leaves of *Spilanthes acmella* Dose: 4.14 mg/20 g BW/day	Glucocorticoid-induced osteoporosis mice	To analyze the effect of *Spilanthes* *acmella* and physical exercise in increasing testosterone and osteoblast cells of femoral’s trabecular glucocorticoid-induced osteoporosis male mice	i. Testosterone levels ii. Bone histology	Combination of *Spilanthes acmella* and exercise increased testosterone level and osteoblast cells compared to osteoporosis group	*Spilanthes acmella* has an additive effect of exercise in protection against glucocorticoid-induced osteoporosis
Pradhan et al., 2021 [31]	Clinical study	SA3X capsules (containing 500 mg of *Spilanthes acmella* extract, standardized to 3.5% spilanthol delivering 17.5 mg spilanthol)	Population-based study: 240 male subjects	To determine the effects of *Spilanthes acmella* on muscle mass	i. Muscle mass assessments: mid upper-arm circumference (MUAC), chest circumference (CC), thigh circumference (TC)	A significant increase in the MUAC	*Spilanthes acmella* may be a potent muscle gainer
Stein et al., 2021 [36]	In vitro and in vivo study	*Acmella oleracea* leaves and flowers extracts; spilanthol Dose: 50–200 μM (in vitro) and 6.2 mg/kg IP (in vivo)	Vascular smooth muscle cells (VSMC) in hyperglycemic media and formalin induced paw edema in rats	To characterize the anti-inflammatory effects of *Acmella oleracea* and spilanthol	In vitro: i. Chymase ii. ROS production In vivo: ii. Paw volume iii. NO level iv. Histology-cellularity	Reduced chymase activity and expression and reduced ROS production Reduced paw edema, NO production, and cell tissue infiltration	*Acmella oleracea* and spilanthol possess significant anti-inflammatory activity
Moro et al., 2021 [37]	In vivo study	Topical application of 20% *Acmella oleracea* leaves and flowers ointment	Rats with partial transection of calcaneal tendon	To analyze the effects of topical application of *Acmella oleracea* ointment (20%) on the repair process of the calcaneal tendon in rats	i. Morphometry ii. Polarization microscopy: birefringence iii. Measurements iv. Biomechanical parameters v. Hydroxyproline quantification	Topical *Acmella oleracea* promoted healing of calcaneal tendon Higher birefringence values and hydroxyproline concentration of collagen in the tendon	Topical *Acmella oleracea* ointment increased the molecular organization and content of collagen, thus presenting a potential application in tendon repair
Barman et al., 2009 [38]	In vivo study	Ethanolic extract of leaves of *Spilanthes acmella* Dose: 500 mg/kg	Carrageenan and Freund’s Complete Adjuvant induced rat paw edema	To evaluate the anti-inflammatory and analgesic activities of *Spilanthes acmella*	i. Paw volume ii. Arthritis index	ELSA (500 mg/kg, p.o) showed significant reduction in paw volume and arthritis score compared to the control group	*Spilanthes acmella* possesses significant anti-inflammatory activity
Indrayani et al., 2024 [39]	In vivo study	*Acmella oleracea* leaves ethanol extract Dose: 200 and 400 mg/kg BW	Monosodium iodate (MIO) induced knee osteoarthritis in rats	To evaluate the potential of *Acmella oleracea* leaves for treatment of osteoarthritis in a rat model	i. Neuropathic pain scores using the Randall Selitto method ii. Interleukin-1β (IL-1β) and tumor necrosis factor-α (TNF-α) levels. iii. Knee joint histology	Reduced pain scores. Lowered IL-1β levels (200 and 400 mg/kg BW). Lowered TNF-α levels (400 mg/kg BW)	*Acmella oleracea* leaf extract can reduce pain and inflammation of osteoarthritis-induced rat joint homogenates
Paul et al., 2016 [40]	In vivo study	*Acmella uliginosa* (AU) (Sw.) Cass. Flower Dose: 417 mg/kg and 833 mg/kg	Rats with model of arthritic paw swelling, Freund’s Complete Adjuvant	To explore the anti-arthritic properties of *Acmella uliginosa*	i. Paw circumference, serum biochemical parameters ii. Gas chromatography/mass spectrometry (GC/MS) analyses	Reduced paw swelling. Increased hemoglobin, serum protein, and albumin levels. Normal creatinine level. GC/MS analyses revealed five anti-inflammatory compounds	Crude flower homogenate of AU contains potential anti-inflammatory compounds, which could be used as an anti-inflammatory/anti-arthritic medication
Rondanelli et al., 2020 [30]	Clinical study	Food-grade lecithin formulation of standardized extracts of *Zingiber officinale* and *Acmella oleracea* Dose: 2 tablets/day for 4 weeks	50 patients with knee osteoarthritis	To evaluate the efficacy of lecithin formulation of standardized extracts of *Zingiber officinale* and *Acmella oleracea* in reducing the pain and inflammation of osteoarthritis	i. Pain intensity by visual analog scale (VAS) ii. WOMAC (Western Ontario and McMaster Universities Arthritis) Index and Tegner Lysholm Knee Scoring iii. Health-related quality of life: 36-Item Short Form Health Survey (SF-36) iv. C-reactive protein (CRP) and erythrocyte sedimentation rate (ESR) v. Body fat composition by dual-energy X-ray absorptiometry (DEXA)	A significant decrease in VAS. Significant improvements in WOMAC, Lysholm, and SF-36 scores. Significant decrease in CRP and ESR, and increase in fat-free mass	The tested formulation seems to be effective in reducing pain and inflammation of osteoarthritis

## Data Availability

Not applicable.

## Abbreviations

The following abbreviations are used in this manuscript:

MSD	Musculoskeletal disorders
PRISMA	Preferred Reporting Items for Systematic Reviews and Meta-Analysis
WHO	World Health Organization
PICOS	Population, Intervention, Comparison/Comparator, Outcomes, and Study
NOS	Newcastle-Ottawa Scale
SYRCLE	Systematic Review Centre for Laboratory Animal Experimentation
NF-κB	Nuclear factor kappa-light-chain-enhancer of activated B cells
MC3T3-E1 cells	Murine calvarial pre-osteoblast cell line
ALP	Alkaline phosphatase
MUAC	Mid upper arm circumference
CC	Chest circumference
TC	Thigh circumference
VSMC	Vascular smooth muscle cells
CFA	Carrageenan and Freund’s Complete Adjuvant
IL-1β	Interleukin-1 beta
TNF-α	Tumor necrosis factor
NO	Nitric oxide
VAS	Visual analog scale
WOMAC	Western Ontario and McMaster Universities Osteoarthritis
SF-36	36-Item Short Form Health Survey
DEXA	Dual-energy X-ray absorptiometry
ESR	Erythrocyte sedimentation rate
CRP	C-reactive protein
Micro-CT	Micro-computed tomography
IP	Intraperitoneally
GCMS	Gas chromatography/mass spectrometry
LCTOFMS	Liquid chromatography time-of-flight mass spectrometry
DPPH	2,2-ediphenyl-1-picrylhydrazyl
ABTS	2,2′-azino-bis (3-ethylbenzothiazoline-6-sulfonic acid)
FRAP	Ferric ion reducing antioxidant potential
BW	Body weight
SA3X	*Spilanthes acmella*
ROS	Reactive oxygen species
ELSA	Ethanolic extract of leaves of *Spilanthes acmella*
MIO	Monosodium iodate
GC/MS	Gas chromatography/mass spectrometry
